# Molecular docking analysis of sphingosine kinase 1 inhibitors for cancer management

**DOI:** 10.6026/97320630019571

**Published:** 2023-05-31

**Authors:** Jameel Barnawi

**Affiliations:** 1 Department of Medical Lab Technology, Prince Fahd Bin Sultan Research chair, Faculty of Applied Medical Sciences, University of Tabuk, Tabuk 71491, Saudi Arabia

**Keywords:** Cancer, sphingosine kinase 1, natural compounds, virtual screening

## Abstract

Sphingosine kinase 1 (SK1) catalyses the conversion of sphingosine to the signalling mediator sphingosine 1-phosphate. This is essential for cell survival and proliferation. SK1 is frequently overexpressed in various cancer types, promoting tumor progression. SK1 has been well documented as a promising target for anticancer therapy. In this study, a virtual screening approach was used to screen a total of 1068 natural compounds, with the aim of identifying potential inhibitors of SK1. The top hit compounds, namely CNP0296172, CNP0368143, CNP0380570, and CNP0290815, were selected based on their strong binding affinity and specificity towards the SK1 binding pocket. Notably, these selected hit compounds exhibited a higher affinity towards the SK1 binding pocket when compared to the positive control compound (PF-543). Furthermore, these compounds were found to meet the necessary drug like criteria, thus rendering them suitable candidates for further experimental validation as potential anticancer agents.

## Background:

Cancer is a prevalent disease that imposes a significant burden on the healthcare system. It is characterized by unregulated cell proliferation caused by genetic mutations, which results in malignant tumors [11]. The incidence and mortality rates of cancer increase exponentially, with an estimated 19.3 million new cases and around 10 million deaths in 2020 [2]. Although conventional treatments such as radiation therapy, chemotherapy, and surgical resection are commonly used to manage cancer, their effectiveness is limited due to cellular resistance [3].

Sphingolipids are important components of cellular membranes, contributing to their structure as well as function [4]. Sphinganine, ceramide, sphingosine, and sphingosine 1-phosphate (S1P) are metabolites that play important roles in a variety of biological processes, including cell proliferation, differentiation, and apoptosis. Furthermore, sphingolipids have been linked to the development of a variety of disorders [5], and they have been shown to affect cancer etiology, progression, angiogenesis, proliferation, therapy resistance, and cell death [16]. Sphingosine kinases (SKs), specifically SK1 and SK2, catalyze the conversion of sphingosine to S1P and play an important role in controlling the balance of ceramides, sphingosine, and S1P [7]. Among these, SK1 has received special attention in cancer research and is considered a bona fide oncogene [8]. A considerable body of evidence suggests that SK1 is present in tumor tissues, with overexpression being observed in the majority of investigations [99,^10^-^11^11].

**Figure 1:**
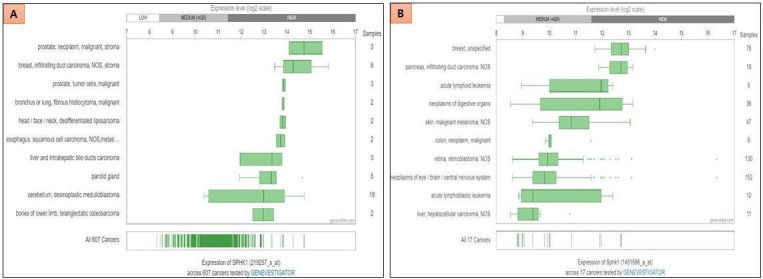
Expression of SK1 in different cancers. Expression analyses performed by Genevisible showing the SK1 expression in human cancer (A) and mouse cancer (B)

Through a comprehensive analysis utilizing the Genevisible web tool (http://genevisible.com), a noteworthy expression of SK1 in cancer cases of both humans and mice has been observed (Figure 1). The results indicate that SK1 exhibits abundant expression in various cancer types, including prostate cancer, malignant neoplasms, infiltrating duct carcinoma of the breast, as well as other types of cancers in humans. Similarly, in mice, SK1 demonstrates high expression levels in breast cancer, pancreatic cancer, and infiltrating duct carcinoma. These findings highlight the potential relevance of SK1 as a target for therapeutic interventions in cancer treatment.

Drug development is a difficult and time-consuming process that encompasses multiple stages. Drug molecules are initially evaluated for their specificities and activity, followed by an assessment of their pharmacokinetics and toxicities later on. Regardless, many medication candidates fail in the latter stages of research due to unfavorable safety and effectiveness difficulties. Significant advances in computational science have recently provided an alternate method for finding potential bioactive compounds that can target specific diseases of interest. These computational tools present a viable path for speeding up the drug discovery process while reducing the high failure rates associated with traditional drug development methods [12]. This study aims to find the novel natural compound SK1 inhibitors to manage the cancers.

## Methods:

## Protein 3D structure preparation:

SK1 was the protein of interest in the study, and its crystal structure (PDB ID: 4V24) complex with PF-543 was obtained from the protein databank [13]. The protein was prepared for virtual screening (VS) by removing the coordinates of heteroatoms and PF-543.

## Compound library preparation:

A collection of 1068 natural compounds ranging in molecular weight from 350 to 450 was obtained from the 'coconut database', a highly regarded and systematically annotated natural compounds database. A filtering process was implemented to ensure the selection of compounds that adhere to Lipinski's rule of five. The compounds were downloaded in '.sdf' format and were prepared after energy minimization with the mmff94 force field. The prepared natural compounds were then saved in the 'pdbqt' format, allowing for VS in subsequent steps.

## Docking protocol validation:

To validate the molecular docking protocol, a redocking experiment with the bound ligand PF-543 in the active pocket of SK1 was conducted. Following this, the initial x-ray crystallographic conformation of PF-543 was aligned with the active pocket. Remarkably, a precise alignment was attained, demonstrating a perfect match between the x-ray crystallographic conformation of PF-543 and the optimal docked pose.

## Virtual screening:

VS are crucial steps in the drug design process, which is used in the study to assess the compounds' affinity for the target SK1 protein. The active site of SK1, in which the inhibitor PF-543 was co-crystallized, was utilized as positive control for the VS of the prepared coconut database library. The binding affinity calculation was performed using PyRx AutoDock VINA, which is an important factor in determining the strength of binding between the compounds and the target protein [14- 15,16]. After that, a comprehensive interaction analysis as well as visual inspections of 2D and 3D interactions was carried out in order to determine the most stable complex based on lower values of binding affinity.

## Result and Discussion:

In our pursuit of discovering natural inhibitors of SK1, a structure-based VS approach was employed. This involved screening a meticulously prepared database of 1068 natural compounds, with PF-543 serving as our positive control. It is important to note that PF-543 is widely recognized as a highly effective inhibitor of SK1. Using PF-543 as a benchmark, the goal was to find promising natural compounds that exhibit similar or better inhibitory properties against SK1.

**Figure 2:**
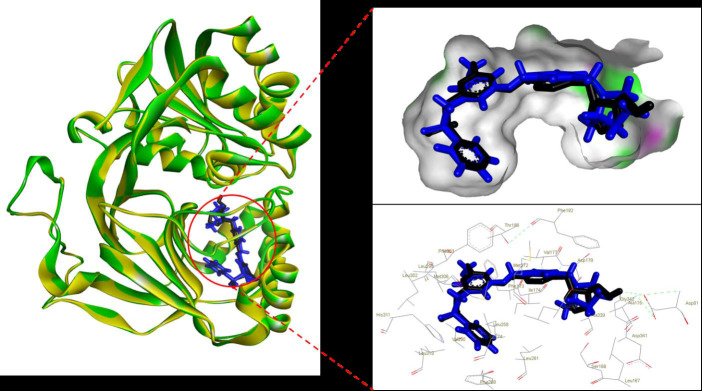
Superimposition of the re-docked PF-543 (blue) with the x-ray structure conformation (black).

To initiate, the docking protocol was validated in order to ensure its accuracy and reliability. Figure 1 Figure 2 demonstrates the excellent alignment between the redocked complex and the originally downloaded complex from the PDB, confirming the robustness of our molecular docking protocol. Following validation, the prepared compound library was subjected to docking-based VS against SK1.

During the screening process, the XYZ coordinates were set to -25.932, 9.521, and -13.531, which were derived from the coordinates of PF-543 in the crystal structure. This strategic choice was used to focus on the specific active site of SK1, increasing the precision and relevance of VS. The best 10 compounds were listed in Table 1 which shows higher binding energy with compare to the control.

**Figure 3:**
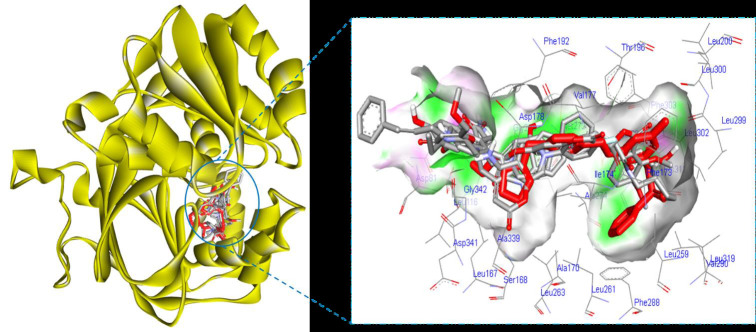
Binding poses of control (red) and best 4 compounds.

This study explored the top four resulted compounds with their detail interaction and other details (Figure 3). Molecular descriptors are numerical descriptions of compounds that capture a variety of the chemical and physical properties of the molecule [17]. In the process of finding new drug molecules, these descriptors play a crucial role. Table 2 describes the different essential descriptors for four finally selected compounds CNP0296172, CNP0368143, CNP0380570, and CNP0290815; and these values indicate them to have well suited for being a lead molecule.

**Figure 4:**
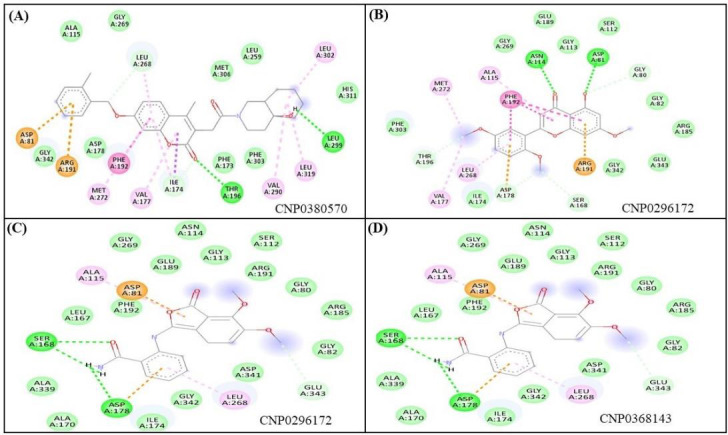
Interacting residues of SK1 protein with CNP0296172, CNP0368143, CNP0380570, and CNP0290815.

**Figure 5:**
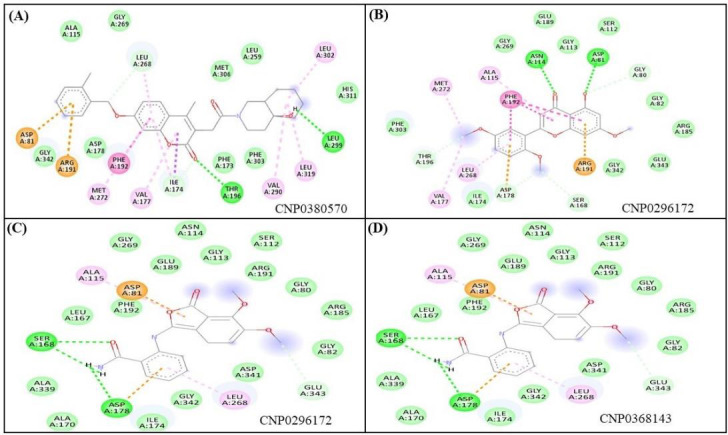
Interacting residues of SK1 protein with positive control PF-543.

CNP0296172, CNP0368143, CNP0380570, and CNP0290815 bind to SK1 protein via hydrogen bonds, Van der Waals force, and other interactions. CNP0380570 interacted with Ala115, Gly269, Leu268, Met306, Leu259, Leu302, His311, Leu299, Leu319, Val290, Phe303, Phe173, Thr196, Ile174, Val177, Phe192, Met272, Asp178, Arg191, Gly342, and Asp81 residues of SK1 protein. Thr196 and Leu299 residues were H-bonded with CNP0380570 (Figure 4AFigure 4A). CNP0296172 interacted with Phe303, Met272, Ala115, Phe192, Gly269, Asn114, Gly113, Glu189, Ser112, Asp81, Gly80, Gly82, Arg185, Glu343, Gly342, Arg191, Ser168, Asp178, Ile174, Leu268, Val177, and Thr196 residues of SK1 protein. Asn114 and Asp81 residues were H-bonded with CNP0296172 (Figure 4B). Next, CNP0368143 interacted with Ser168, Leu167, Phe192, Asp81, Ala115, Glu189, Gly269, Asn114, Gly113, Ser112, Arg191, Gly80, Arg185, Gly82, Glu343, Asp341, Leu268, Gly342, Ile174, Asp178, Ala170, and Ala339 residues of SK1 protein. Ser168 and Asp178 residues were H-bonded with CNP0368143 (Figure 4C). Furthermore, CNP0290815 interacted with Asp178, Thr196, Met306, Phe303, Phe173, Val177, Ile174, Met272, Leu268, Gly269, Phe192, Asp81, Glu189, Leu167, Gly113, Ala115, Asn114, Ser112, Arg191, Gly80, Gly82, Gly342, Asp341, and Ser168 residues of SK1 protein. Leu268 residue was H-bonded with CNP0290815 (Figure 4D). PF-543 is a novel competitive inhibitor of sphingosine [18]. The standard drug PF-543 was found to interact with Asp81, Leu167, Gly342, Ser168, Asp178, Phe192, Val177, Thr196, Phe173, Leu299, Leu200, Phe303, Leu302, Leu319, His311, Phe288, Leu261, Val290, Ala274, Leu259, Ile174, Met306, Met272, Leu268, and Ala115 residues of SK1 protein (Figure 5). Notably, the identified hit compounds CNP0296172, CNP0368143, CNP0380570, and CNP0290815 were observed to interact with these SK1 residues (Figure 4A-D, & Figure 5), implying that these compounds bind to the same binding pocket of SK1 protein as PF-543.

Natural products are a useful source of bioactive chemicals with medicinal potential due to their high chemical variety. Significant efforts have been made in recent decades to find and isolate novel natural products from a range of sources, including microbes, plants, and other species. These natural chemicals have been intensively studied for their anticancer qualities and underlying mechanisms of action, which has resulted in the invention of various anti-cancer medications [19]. Surprisingly, it is projected that between 1981 to 2019 and nearly 25% of newly authorized anti-cancer medications were derived from natural ingredients [20,2121].

## Conclusion:

This study shows the molecular interactions between natural compounds and the SK1 protein, with a focus on CNP0296172, CNP0368143, CNP0380570, and CNP0290815, which demonstrated a strong binding affinity for SK1. Furthermore, these compounds were found to meet the essential drug like criteria, making them suitable candidates for additional experimental validation as potential anticancer agents.

## Figures and Tables

**Table 1:** Best 10 screened hits and their binding affinity.

**Natural compound**	**Binding affinity (kcal/mol)**
CNP0296172	-10.9
CNP0368143	-10.7
CNP0290815	-10.5
CNP0380570	-10.5
CNP0417010	-10.5
CNP0306610	-10.4
CNP0395063	-10.3
CNP0143455	-10.2
CNP0328107	-10.2
PF 450	-10.1

**Table 2:** Molecular descriptor of selected four compounds

**Molecular Descriptors**	**compound name**			
	**CNP0296172**	**CNP0368143**	**CNP0380570**	**CNP0290815**
NP-likeness score	0.85	0.21	0.96	1.12
Alogp	-0.49	-0.45	2.75	-0.26
Alogp2	0.24	0.2	7.58	0.07
Apol	47.1607	46.7987	78.1542	52.8163
Bpol	26.1133	25.1553	43.8038	30.2157
ECID	434	435	1115	624
FmfDescriptor	0.6667	0.6667	0.8571	0.9259
Fsp3	0.1667	0.1765	0.4483	0.35
FCD	1212.06	1212.07	3994.06	1699.07
Petitjean Number	0.4545	0.4545	0.4737	0.4615
Lipinski Rule Of 5 Failures	0	0	0	0
WienerPathNumber	1269	1300	4236	1888
Xlogp	1.752	0.787	3.376	1.408
ZagrebIndex	126	126	192	152
TopoPSA	74.22	99.88	76.07	83.45
(ECID=Eccentric Connectivity Index Descriptor; FCD=Fragment Complexity Descriptor)
